# Digital Leadership, Information Entropy, and Stock Price Volatility: Evidence from CEO Social Media Behavior

**DOI:** 10.3390/e28020200

**Published:** 2026-02-10

**Authors:** Yutong Zou, Jingqian Tian, Yunfan Zhang, Guangping Shi, Xiao Cai

**Affiliations:** 1 Department of FinTech, Nanjing University of Information Science and Technology, Nanjing 210044, China; 202363050013@nuist.edu.cn (Y.Z.); 202363050018@nuist.edu.cn (Y.Z.); 2 Laboratory of Philosophy and Social Sciences at Universities in Jiangsu Province-Fintech and Big Data Laboratory of Southeast University, Southeast University, Nanjing 211189, China; 3 School of Computational Economics, Henan University of Economics and Law, Zhengzhou 410005, China; shigp1210@126.com; 4 Research Center of Applied Electromagnetics, Nanjing University of Information Science and Technology, Nanjing 210044, China; caixiao@nuist.edu.cn

**Keywords:** digital leadership, information entropy, CEO social media behavior, deep learning, stock price volatility

## Abstract

In the digital economy, social media has become a critical channel through which corporate executives communicate with investors, thereby influencing market expectations and price dynamics. This study examines how CEO social media behavior affects stock price volatility from an information-theoretic perspective combined with deep learning methods. Using Lei Jun (Xiaomi) and Elon Musk (Tesla) as contrasting cases, we analyze executive communication under transactional and transformational leadership styles. Emotional tone, thematic alignment, and diffusion intensity are extracted using BERT and LDA, and incorporated into a Long Short-Term Memory (LSTM) model to forecast short-term stock price movements. To interpret the mechanism behind the predictive results, we introduce a novel metric: Semantic Resonance Dissipation Entropy (SRE). Derived from Kullback–Leibler divergence, this indicator measures the informational friction between executive semantic output and market attention. The empirical analysis shows that incorporating these high-dimensional semantic features significantly improves volatility prediction. Moreover, leadership style is closely associated with distinct entropic regimes: Transactional leadership corresponds to relatively stable semantic patterns and low entropy, whereas transformational leadership is associated with higher entropy and greater semantic dispersion. Following Musk’s acquisition of Twitter, the previously unstable information environment evolved into a persistent structural factor priced by the market. These findings suggest that the economic impact of digital leadership depends on limiting information dissipation to ensure signal clarity in financial markets.

## 1. Introduction

In financial markets, asset pricing and volatility prediction are not merely technical exercises but reflections of how information is generated, transmitted, and cognitively absorbed by investors. Over the past decade, this information infrastructure has undergone a profound transformation. Corporate disclosures that were once primarily conveyed through earnings calls or regulatory filings are now increasingly supplemented, and in some cases overshadowed, by executives’ real-time communication on social media platforms. A single post by a high-profile CEO can instantaneously trigger surges in trading volume, sharp volatility spikes, or rapid reversals in investor sentiment. Despite the growing prevalence of such episodes, the mechanisms through which unstructured and informal executive signals translate into market dynamics remain insufficiently understood.

This ambiguity revives a long-standing theoretical tension in finance. According to the Efficient Market Hypothesis proposed by Fama [1], asset prices fully and instantaneously incorporate all publicly available information, implying that informal narratives or discretionary disclosures should not generate systematic predictability. In contrast, behavioral finance emphasizes that investors process information through cognitive filters shaped by limited attention, heuristics, and emotional responses. Consequently, even non-fundamental signals may induce transient mispricing and volatility clustering [2]. Executive communication on social media lies precisely at the intersection of these perspectives: it is public and rapidly disseminated, yet inherently subjective, context-dependent, and susceptible to interpretive distortion.

From a behavioral standpoint, Upper Echelons Theory provides an important micro-level foundation by arguing that executives’ cognitive frames and value systems materially influence organizational outcomes [3]. When CEOs engage directly with the public through social media, they act not only as strategic decision makers but also as dominant information broadcasters. Their personal narratives, emotional tone, and topical emphasis actively shape patterns of market attention. Prior empirical studies document that CEO online behavior, including posting frequency and sentiment orientation, can meaningfully affect investor behavior and firm valuation [4]. However, much of the existing literature implicitly assumes that the semantic content of executive communication is transmitted to the market without friction, treating such signals as static inputs rather than as dynamic messages that may be amplified, distorted, or dissipated during diffusion.

Modeling this transmission process poses substantial methodological challenges. Conventional econometric approaches, such as linear regression and classical time series models including ARIMA, are poorly suited to the high-dimensional, nonlinear, and rapidly evolving nature of social media text [5]. Recent advances in deep learning, particularly Long Short Term Memory networks, have substantially improved predictive performance by capturing complex temporal dependencies in financial data [6]. Nevertheless, these models often operate as opaque black boxes, offering limited insight into why certain executive messages enhance market predictability while others inject noise. Previous studies have proposed hybrid frameworks integrating sentiment indicators with price dynamics [7]. However, existing literature has yet to provide a principle-based quantitative measurement method for assessing the structural transmission pathways between the semantic signals conveyed by CEO intentions and the collective cognitive responses of the market.

Several imbalances further characterize the existing research. First, most studies rely on isolated features such as sentiment polarity or message volume, overlooking the multidimensional interaction between emotion, thematic content, and social diffusion. Second, relatively little attention has been paid to leadership heterogeneity, particularly the distinction between transactional and transformational styles, and how such differences moderate the effectiveness of AI-driven prediction models. Third, and most critically, current work lacks a unified theoretical framework that integrates information-theoretic concepts with deep learning to explain why prediction models succeed or fail under different leadership regimes.

To address these limitations, this study develops an interdisciplinary framework that integrates deep learning with an information-theoretic perspective. The primary contributions of this paper can be summarized as follows:

- A multidimensional Sentiment Topic Virality feature system is constructed to characterize the semantic structure of CEO social media communication. By applying BERT-based sentiment embeddings together with topic modeling techniques, these features are embedded into an LSTM architecture, enabling the model to capture the nonlinear relationship between executive communication signals and short-term stock price volatility.
- Semantic Resonance Dissipation Entropy is creatively proposed in this study as a novel information theoretic metric, grounded in Shannon’s information entropy and operationalized via Kullback–Leibler divergence [8]. It quantifies the distributional divergence between a CEO’s semantic output vector and the market’s attention feedback vector, thereby serving as a proxy for informational friction during the transmission and diffusion of leadership signals.
- Using Lei Jun of Xiaomi and Elon Musk of Tesla as representative cases, we provide comparative empirical evidence on how leadership style is associated with distinct market entropy patterns. The results indicate that transactional leadership is generally associated with lower entropy and higher predictability, whereas transformational leadership tends to generate higher entropy and greater informational noise. Moreover, we document a structural shift in Musk’s market influence following his platform acquisition, during which the entropy price relationship evolves from chaotic randomness toward a more stable inverse suppression mechanism.

The remainder of this paper is organized as follows. Section 2 reviews the related literature. Section 3 presents the theoretical framework. Section 4 details the methodology for feature extraction and entropy construction. Section 5 reports the empirical results and discussion. Section 6 concludes the study.

## 2. Literature Review

### 2.1. Research on CEO Digital Leadership

With the accelerating evolution of the digital economy, CEOs’ digital leadership is increasingly recognized as a core capability that drives organizational transformation and supports sustained competitive advantage. Digital transformation has reshaped firms’ operational architectures and has imposed higher demands on the competence profiles and role positioning of top executives. Petry characterizes digital leadership as a critical capability that enables organizations to respond to highly uncertain and complex environments through agile decision-making and collective intelligence, while effectively leveraging emerging digital technologies [9]. Executives with strong digital leadership are expected to rapidly learn and adapt to technological change, coordinate and integrate internal resources, and support firms in coping with fast-moving external dynamics. Araujo et al. further emphasize that digital leadership extends beyond the adoption of new technologies. Instead, it involves a deep understanding of organizational structures, business processes, and business models, alongside the capacity to promote systemic transformation [10].

The formation of digital leadership is shaped by multiple reinforcing factors. Cortellazzo et al. argue that the rapid development of big data analytics, artificial intelligence, and cloud computing requires leaders to continuously update their knowledge base and skill portfolio [11]. At the same time, organizational culture and structural arrangements play an important enabling role: open, flexible, and innovation-oriented cultures are conducive to the development of digital leadership, whereas rigid and closed structures may constrain its effective realization. Kane et al. show that, while digital leadership retains several characteristics of traditional leadership, it differs substantially in terms of technological sensitivity, innovation orientation, and cross-domain collaboration [12]. Empirical evidence from Wang et al. indicates that CEOs’ digital leadership is positively associated with firms’ digital transformation progress and organizational performance [13]. Leaders with strong digital leadership typically exhibit heightened technological foresight and market awareness and foster cross-functional collaboration and innovative practices through effective communication and coordination.

A growing body of research has documented the substantial influence of digital leadership on operational outcomes and strategic performance. Araujo et al. find that firms with stronger digital capabilities tend to outperform peers in market share, customer satisfaction, and innovation capacity [10]. Digital leaders enhance organizational efficiency through process optimization and resource reconfiguration, thereby strengthening overall competitiveness. In parallel, CEOs’ personal branding has increasingly become an integral component of digital leadership. Variations in leadership styles expressed in digital contexts may generate differentiated psychological contract effects among stakeholders [8]. Moreover, digital leadership contributes to the cultivation of open, collaborative, and empowering organizational cultures, improving employee engagement and satisfaction and supporting talent attraction and retention initiatives [11].

Taken together, CEOs’ digital leadership plays a pivotal role in driving firms’ digital transformation, and their multi-dimensional skills and adaptive capabilities constitute an important foundation for achieving strategic objectives and improving firm performance. Future research may further investigate how digital leadership manifests across different industries and cultural contexts, as well as the mechanisms through which it affects organizational transformation and market outcomes.

### 2.2. Research on Volatility

Stock price volatility represents a central manifestation of risk and uncertainty in financial markets and has long been a focal topic in both academic research and industry practice. Mayhew provides a systematic review of the principal approaches to computing implied volatility and highlights its foundational role in risk management, derivatives pricing, and asset allocation [14]. Modeling work by Diebold and Lopez further documents pronounced volatility clustering and conditional heteroskedasticity in equity returns [15], while Jones, Kaul, and Lipson show that volatility is positively associated with trading frequency, indicating that market activity intensity constitutes an important determinant of price fluctuations [16].

Beyond macro-level market conditions, the behavior and organizational role of CEOs—as key actors in corporate governance—have also been shown to exert a significant influence on stock price volatility. Clayton et al. report that CEO turnover is typically followed by a marked increase in volatility [17], reflecting investors’ reassessment of strategic orientation and future operating prospects under new leadership. Extending this perspective, Gu et al. demonstrate that the degree of CEO power concentration affects managerial risk-taking preferences: more powerful CEOs tend to reduce firm-specific risk exposures, thereby lowering their personal risk-bearing costs [18]. They also find that voluntary CEO departures are associated with higher levels of idiosyncratic volatility compared with involuntary exits, suggesting heightened market attention to governance restructuring and potential strategic adjustment [18].

In terms of leadership style and information disclosure, Taylor et al. show that CEOs’ communication strategies can influence stock price volatility by shaping information transparency and expectation formation in financial markets [19]. This finding resonates with evolving leadership requirements in the digital environment. Kane et al. argue that, in digital contexts, executives must integrate traditional managerial capabilities with digital competencies in order to maintain organizational stability and agility amid rapid technological change and accelerated information diffusion [12]. Accordingly, CEOs’ digital leadership may not only affect strategic implementation and internal coordination but may also shape external market expectations, thereby exerting an indirect yet potentially important influence on volatility dynamics.

Despite substantial progress in examining the relationship between CEOs’ leadership attributes, governance structures, and stock price volatility, existing studies remain subject to several important limitations. Much of the literature conceptualizes executive communication either as a static disclosure event or as a background governance characteristic, devoting limited attention to the dynamic information transmission process through which managerial signals are generated, disseminated, and interpreted by market participants. Existing studies rarely consider the informational uncertainty inherent in executive signals, which can be formally quantified using Shannon’s information entropy [20], thereby overlooking how variability in message content and reception contributes to market volatility. In addition, although digital leadership has attracted increasing scholarly interest, prior research has predominantly focused on its implications for internal organizational performance and strategic transformation, while its external market effects, particularly in high-frequency information environments such as social media, remain insufficiently explored. Empirical approaches in this strand of research often rely on coarse measures of communication intensity or visibility, without systematically distinguishing the informational content, emotional orientation, and diffusion effectiveness of executive messages.

To address these gaps, this study conceptualizes CEOs’ social media activity as a structured information signal embedded in a noisy and attention-constrained market environment. Drawing jointly on information diffusion theory [21], Shannon’s information theory [20], and leadership style theory, we develop a mechanism-based framework to examine how CEO digital leadership shapes stock price dynamics through the generation, diffusion, and reception of executive signals, with the impact mediated by variations in information entropy. Specifically, the paper proposes a multi-dimensional Sentiment Topic Virality framework to capture how the emotional tone, thematic focus, and social penetration of CEO communications jointly influence the informational entropy of market-relevant signals, investor attention, expectation dispersion, and short-term stock price volatility. By integrating these dimensions into a dynamic prediction model, the study advances a micro-behavioral perspective on volatility formation and provides empirical evidence on how heterogeneous leadership styles produce differentiated market responses through entropy-mediated information effects.

## 3. Materials and Methods

### 3.1. Theoretical Basis

This study is grounded in a mechanism-oriented perspective, conceptualizing CEOs’ social media behavior as a sequential information transmission process that links executive communication to stock price dynamics. Rather than treating CEO posts as isolated disclosure events, the analysis situates them within a dynamic chain comprising signal generation, information diffusion, market reception, and price response. Three complementary theoretical frameworks—information diffusion theory, Shannon’s information theory, and leadership style theory—are integrated to explain distinct stages of this process.

At the initial stage, CEOs’ social media posts function as deliberate strategic signals released into a digitally mediated information environment. Information diffusion theory emphasizes that propagation in social networks is inherently nonlinear and feedback-driven, shaped by network structure, propagation speed, and audience interaction [21]. In environments characterized by dense connectivity and immediacy, individual posts can rapidly reach a broad audience, yet the effective transmission of information depends on whether these signals are successfully received, interpreted, and acted upon by market participants. Interaction metrics, such as reposts, comments, and likes, provide observable proxies for the extent and intensity of information diffusion.

Following dissemination, the impact of executive signals on market perception is framed using Shannon’s information theory [20]. In this context, stock price fluctuations are interpreted as reflecting the degree of alignment or mismatch between the information emitted by the CEO and the attention and interpretation of the market. When signals are poorly received or fail to match market focus, informational disorder arises, which manifests as increased uncertainty and volatility in stock prices. Conversely, when the CEO’s communications are effectively perceived and interpreted by the market, alignment is achieved, mitigating uncertainty and stabilizing price movements. This stage captures the dynamic process through which the clarity, timing, and resonance of executive messages influence market responses.

Leadership style theory is then introduced to account for heterogeneity in signal generation and diffusion effectiveness. Transactional and service-oriented leaders, in contrast, may focus on operational transparency and incremental guidance, leading to more moderate yet consistent impacts on market interpretation and price dynamics.

**Figure 1:**
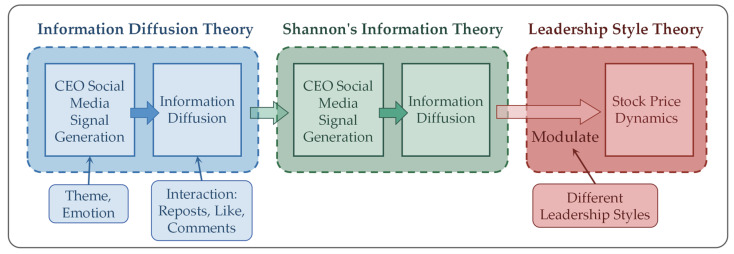
Mechanism of CEO social media information transmission.

Accordingly, this study operationalizes CEO social media behavior along three interrelated dimensions: the thematic relevance of content, the emotional orientation of messages, and the intensity of public engagement. By integrating these dimensions, the proposed framework captures the full mechanism through which CEO digital leadership shapes information diffusion, generates varying degrees of market alignment, and ultimately influences stock price dynamics. The transmission pathway is shown in Figure 1.

### 3.2. Technological Path

**Figure 2:**
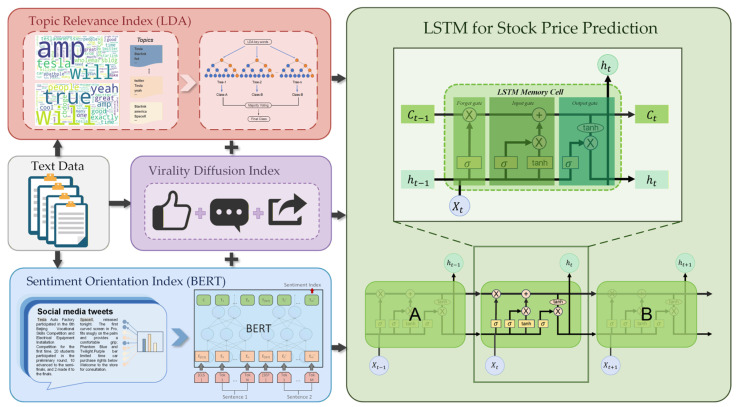
Research Framework.

Following the establishment of a three-dimensional index system consisting of a Sentiment Orientation Index, a Topic Relevance Index, and a Virality Diffusion Index, this study applies tailored quantitative procedures to construct each index. The research framework diagram is shown in Figure 2.

For the Sentiment Orientation Index, sentiment features of CEOs’ social media posts are extracted using a BERT-based classification framework. BERT is a pre-trained language model developed for natural language processing tasks, whose underlying architecture is built upon a multi-layer bidirectional Transformer encoder. Through contextualized representation learning, BERT captures deep semantic dependencies in text. Compared with traditional unidirectional language models, its primary advantage lies in its bidirectional context modeling capability, enabling the model to incorporate both preceding and succeeding contextual information when encoding each token, thereby improving the accuracy of semantic interpretation and emotional attribution. To obtain a continuous measure of sentiment characteristics, sentiment polarity scores are normalized to the interval [0, 1], where 0 denotes fully negative sentiment and 1 denotes fully positive sentiment. Based on the predicted sentiment scores for each post, a Sentiment Orientation Index is constructed to characterize the emotional tendency embedded in CEOs’ posts.

For the Topic Relevance Index, this study employs Latent Dirichlet Allocation (LDA) to model the textual content of CEO posts. As a classical generative probabilistic model [22], LDA identifies latent thematic structures from large-scale unstructured text corpora and has been widely applied in financial text mining to trace shifts in market narratives and attention dynamics [23]. LDA is built upon a three-layer Bayesian structure consisting of documents, topics, and terms, and explains word co-occurrence patterns through latent topic distributions. It assumes that each document is generated from a mixture of multiple latent topics, and that each topic corresponds to a probability distribution over terms, thereby revealing hidden semantic structures across the text collection.

To determine the optimal number of topics K, both Perplexity and Topic Coherence are adopted as joint evaluation criteria. From an information-theoretic perspective, perplexity can be interpreted as an exponential form of Shannon entropy, reflecting the degree of uncertainty in the model’s generative probability distribution. Lower perplexity implies lower informational entropy and a more certain grasp of latent semantic patterns. However, minimizing perplexity alone may induce overfitting. Therefore, Topic Coherence is incorporated to evaluate the semantic relatedness of top terms within each topic. By jointly considering information uncertainty and semantic interpretability, the optimal topic number is selected, and the Top 30 topic keywords associated with each CEO are extracted. These keywords are subsequently mapped to Baidu Index and Google Trends to obtain corresponding search intensity indicators, which serve as the Topic Relevance Index. This index captures whether the focal themes emphasized in CEOs’ posts diffuse into the broader information environment and trigger public attention responses.

For the construction of the Virality Diffusion Index, three interaction indicators are selected for each post: the number of reposts, comments, and likes. These indicators reflect immediate market feedback intensity from different behavioral dimensions and provide an integrated measure of how the public perceives and evaluates firms’ strategic messages and value signals transmitted through CEOs’ social media activity.

After constructing the three-dimensional indicator framework, this study applies a Long Short-Term Memory (LSTM) model to forecast stock price time series. LSTM is a baseline model for sequential prediction and is well-suited to financial time series characterized by non-stationarity, nonlinearity, and long-memory behavior [24]. Empirical evidence from Fischer and Krauss shows that LSTM networks outperform Random Forests and conventional deep neural networks in capturing long-term dependency structures in equity markets [6]. As an improved form of recurrent neural network (RNN), LSTM incorporates input, forget, and output gates to mitigate gradient vanishing and explosion problems that arise in long-horizon learning. In this study, an LSTM-based architecture named DeepRegressionLSTM is implemented under the PyTorch v2.4.0 framework. The network integrates LSTM layers for dependency modeling and fully connected layers to map hidden states to final prediction outputs, enabling stock price regression forecasting.

However, given the pronounced heterogeneity in information diffusion mechanisms associated with different leadership styles, a single deep learning model often struggles to explain variations in predictive performance across samples. To further open the “black box” of the forecasting model and investigate how executive behavior moderates market uncertainty, this study innovatively constructs Semantic Resonance Dissipation Entropy (SRE) within the framework of information entropy theory. The SRE metric projects the CEO’s semantic output vector and the market’s attention feedback vector into a unified probabilistic space. The former is constructed from BERT-based sentiment representations and weighted word-frequency features, while the latter is derived from search trend data. The distributional divergence between the two is then quantified using relative entropy (Kullback–Leibler divergence). Conceptually, Semantic Resonance Dissipation Entropy captures the degree of spatiotemporal misalignment between CEO signal transmission and market cognitive processing. Lower entropy indicates strong resonance between semantic content and market attention, reflecting low friction and high certainty in information transmission. Higher entropy, by contrast, signals a decoupling between semantic output and market attention, implying increased dissipation and noise during the information diffusion process. By introducing this interdisciplinary metric at the intersection of physics and information theory, this study seeks to explain, from a microstructural perspective, the deeper mechanisms through which CEO social media behavior shapes stock price predictability.

Within this analytical framework, a comparative case study approach is adopted, focusing on two corporate executives with distinctly different leadership styles. Elon Musk, Chief Executive Officer of Tesla and SpaceX, is selected as a representative case of transformational leadership. Musk maintains a high level of activity on the X platform (formerly Twitter), with posts characterized by visionary narratives, technological radicalism, and a highly personalized tone, reflecting strong transformational attributes. As a contrasting case, Lei Jun, Chief Executive Officer of Xiaomi Group, is selected to represent a transactional leadership style. Known for his pragmatic and steady managerial philosophy, Lei Jun engages in frequent interactions on platforms such as Weibo, primarily centered on product iteration and user feedback, exhibiting typical transactional and service-oriented leadership characteristics.

Based on the above methodological design and sample selection, this study constructs a social media content analysis framework to perform text mining and quantitative analysis of the public posts of both CEOs. Combined with capital market data, the analysis focuses on three core research questions. First, whether the multidimensional characteristics of CEO social media posts—encompassing sentiment, topic orientation, and social penetration—exert a direct influence on short-term stock price fluctuations. Second, whether CEOs with different leadership styles, through heterogeneous information diffusion behaviors on social media, exert differentiated effects on stock price forecasting performance via distinct market reaction mechanisms. Third, whether Semantic Resonance Dissipation Entropy can effectively account for variations in the predictive performance of stock price forecasting models across different leadership styles. Through addressing these questions, this study aims to reveal the economic consequences of executive information dissemination behavior in the social media era, providing new empirical evidence and theoretical insights for corporate governance research and investment decision-making in digital financial markets.

## 4. Methodology

### 4.1. Construction of Sentiment–Topic–Virality (STV) Indicators

#### 4.1.1. Sentiment Orientation Index

To capture the latent market sentiment embedded in CEOs’ social media posts, this study constructs a Sentiment Orientation Index using a BERT-based framework. BERT, introduced by Devlin et al., employs a multi-layer bidirectional Transformer encoder architecture and leverages a masked language modeling pretraining objective to generate dynamic representations of deep syntactic structures and contextual semantic dependencies within text [25]. Unlike traditional approaches, such as the static financial sentiment dictionaries developed by Loughran and McDonald [26], BERT overcomes the limitations of lexical polysemy. It exhibits superior performance in detecting implicit sarcasm, metaphorical expressions, and domain-specific nonlinear linguistic patterns in financial contexts. As a result, BERT substantially improves both the accuracy and robustness of sentiment classification tasks.

In the financial domain, pre-trained BERT-based models, such as FinBERT, have been demonstrated to substantially enhance the accuracy of sentiment classification on micro-level financial texts and provide an appropriate baseline for constructing a sentiment orientation index [27]. Cross-country analyses further indicate that sentiment indicators derived from Twitter exhibit significant associations with stock returns and trading activity across multiple markets, supporting the integration of social media sentiment into predictive modeling frameworks [28]. Drawing on this methodological framework, we explicitly model the sentiment generation process.

Let the input post sequence be defined as $S_{j}=\{w_{1},w_{2},\ldots ,w_{n}\}$. where n denotes the sequence length. The BERT encoder maps this sequence into deep contextual embeddings. We utilize the vector corresponding to the special classification token (CLS), denoted as $h_{\mathrm{CLS},j}\in {\mathbb{R}}^{d}$, to represent the aggregate semantic information of the entire post, as formally defined in (1):

(1) $$h_{CLS,j}{=\mathrm{BERT}(S}_{j}).$$

Subsequently, to transform this high-dimensional semantic vector into a scalar sentiment metric, $h_{CLS,j}$ is passed through a fully connected layer followed by a Softmax activation function. This projects the semantic features onto a two-dimensional probability distribution:

(2) $$y_{j}=\mathrm{Softmax}(W_{s}h_{\mathrm{CLS},j}+b_{s}),$$

where $W_{s}\in {\mathbb{R}}^{2\times d}$ and $b_{s}\in {\mathbb{R}}^{2}$ are trainable parameters, and $y_{j}=[y_{j}^{\mathrm{neg}},y_{j}^{\mathrm{pos}}]$ represents the output probability vector.

Instead of using a discrete binary label (0 or 1), we extract the probability associated with the positive class as a continuous proxy for sentiment intensity. We define the sentiment score $p_{j}$ for post j as:

(3) $$p_{j}=y_{j}^{\mathrm{pos}}\in [0,1],$$

here, a $p_{j}$ value closer to 1 indicates strong positive sentiment, while a value closer to 0 indicates strong negative sentiment. Finally, the daily Sentiment Orientation Index ($E_{t}$) is constructed by averaging these continuous sentiment scores across all posts $N_{t}$ published on day t, as shown in (4):

(4) $$E_{t}=\frac{1}{N_{t}}\sum_{j=1}^{N_{t}}p_{j}.$$

#### 4.1.2. Topic Relevance Index (LDA)

For the Topic Relevance Index, this study employs Latent Dirichlet Allocation (LDA) to model the textual content of CEO posts.

Based on Shannon’s information theory, this paper employs perplexity as an indicator for measuring the goodness-of-fit of a model’s probability distribution. Mathematically speaking, perplexity is an exponential mapping of the model’s cross-entropy on the test set. We define the model’s average log-likelihood H(D) for the document set D as shown in (5):

(5) $$H(D)=-\frac{\sum_{d=1}^{M}\log p(w_{d})}{\sum_{d=1}^{M}N_{d}},$$

where M denotes the total number of documents, $w_{d}$ represents the word vectors within document d, and $N_{d}$ indicates the length of document d. Based on this, the functional relationship between perplexity and entropy is given by (6):

(6) Perplexity=exp{H(D)}.

A lower perplexity indicates lower information entropy, reflecting a higher certainty of the model in predicting the latent semantic structure. However, solely minimizing entropy may lead to model overfitting, resulting in fragmented topics that are difficult for humans to interpret. To address this, Topic Coherence is introduced as a regularization constraint on semantic quality. Topic coherence measures the semantic similarity among high-frequency words within a topic, and is formally defined as follows:

(7) $$C_{TC}=\sum_{i<j}\log\frac{D(\nu_{i},\nu_{j})+\epsilon }{D(\nu_{j})},$$

where $D(\nu_{i},\nu_{j})$ denotes the joint frequency of words $v_{i}$ and $v_{j}$. occurring within the document collection, $D(\nu_{j})$ represents the word’s occurrence frequency, and ϵ is the smoothing term.

Based on the optimal number of topics, this paper extracts the top 30 high-frequency keyword sets $K=\{k_{1},k_{2},\ldots ,k_{30}\}$ associated with each latent theme. For each keyword $k_{i}$, the paper obtains its search engine search index $SI_{t}^{\left(i\right)}$ on day t. Consequently, the topic-related features are formally constructed as a 30-dimensional vector $T_{t}$, as shown in (8):

(8) $$T_{t}={\left[SI_{t}^{\left(1\right)},SI_{t}^{\left(2\right)},\ldots ,SI_{t}^{\left(30\right)}\right]}^{⊤}.$$

#### 4.1.3. Virality Diffusion Index

To construct a robust social media engagement framework, this study selects three core metrics—repost volume, comment count, and like count—to quantitatively analyze the posts of Lei Jun and Elon Musk. Repost volume captures the breadth of diffusion, reflecting users’ endorsement of the content and their propensity to actively propagate it within the network. Comment count signifies the depth of engagement and deliberation, encompassing diverse feedback—ranging from positive evaluations to critical scrutiny—thereby serving as a direct manifestation of user sentiment and value judgments. Finally, like count represents an immediate endorsement, offering an intuitive proxy for positive sentiment toward the CEO’s viewpoints, corporate developments, or product-related updates.

To eliminate the impact of daily fluctuations in post volume, we calculate the daily average of each metric. The social penetration feature vector $S_{t}$ for day t is defined by (9):

(9) $$S_{t}={\left[\begin{array}{c} {\bar{R}}_{t},{\bar{C}}_{t},{\bar{L}}_{t} \end{array}\right]}^{⊤}.$$

Among these, the daily average values for likes, comments, and shares are calculated in (10):

(10) $${\bar{R}}_{t}=\frac{1}{N_{t}}\sum_{j=1}^{N_{t}}r_{j},{\bar{C}}_{t}=\frac{1}{N_{t}}\sum_{j=1}^{N_{t}}c_{j},\bar{L_{t}}=\frac{1}{N_{t}}\sum_{j=1}^{N_{t}}l_{j},$$

$r_{j}{,c}_{j}{,l}_{j}$ denote the number of reposts, comments, and likes, respectively, for the j-th post on day t.

### 4.2. DeepRegressionLSTM for Stock Price Prediction

Existing studies suggest that incorporating multidimensional features into time-series forecasting frameworks can enhance model stability and predictive accuracy [29]. Following this line of research, this study integrates three dimensions of social media–derived indicators: the Sentiment Orientation Index constructed using BERT, the Topic Relevance Index extracted via the LDA model, and the Virality Diffusion Index derived from post interaction data.

This study applies a Long Short-Term Memory (LSTM) model to forecast stock price time series.

With regard to the model input configuration, this paper aligns the metrics across the aforementioned three dimensions temporally and concatenates them to form the model’s input feature vector $X_{t}$. At time step t, the definition of the input vector is given by (11):

(11) $$X_{t}=E_{t}⊕T_{t}⊕S_{t}=\left[E_{t},SI_{t}^{\left(1\right)},\ldots ,SI_{t}^{\left(30\right)},{\bar{R}}_{t},{\bar{C}}_{t},{\bar{L}}_{t}\right],$$

where ⊕ denotes vector concatenation. The LSTM unit regulates the flow of information via the input gate ($i_{t}$), forget gate ($f_{t}$), and output gate ($o_{t}$). Let $X_{t}$ denote the input feature vector at time step t. The state update equations are presented in (12):

(12) $$f_{t}=\sigma (W_{f}\cdot [h_{t-1},X_{t}]+b_{f}),\;i_{t}=\sigma {\left(W_{i}\cdot [h_{t-1},X_{t}]+b_{i}\right)}_{t},\;{\tilde{C}}_{t}=\tanh(W_{C}\cdot [h_{t-1},X_{t}]+b_{C}),\;C_{t}=f_{t}⊙C_{t-1}+i_{t}⊙\tilde{C},\;o_{t}=\sigma (W_{o}\cdot [h_{t-1},X_{t}]+b_{o}),\;h_{t}=o_{t}⊙\tanh(C_{t}),$$

where σ denotes the Sigmoid activation function, ⊙ represents the Hadamard product, and $h_{t}$ denotes the hidden layer state. Ultimately, the hidden state $h_{t}$ is mapped through a fully connected layer to the predicted stock closing price ${\hat{y}}_{t+1}$, which is output via the fully connected layer, as shown in (13):

(13) $${\hat{y}}_{t+1}=W_{dense}h_{t}+b_{dense}.$$

Mean Absolute Error (MAE) is adopted as the loss function to optimize the model parameters, as defined in (14):

(14) $$L=\frac{1}{N}\sum_{k=1}^{N}|y_{k}-{\hat{y}}_{k}|.$$

### 4.3. Construction of Semantic Resonance Dissipation Entropy

The construction of the Semantic Resonance Dissipation Entropy (SRE) is grounded in Shannon’s classical communication model, which conceptualizes information transmission as a process involving a source, a channel, and a receiver. In the present context, the CEO functions as the information source, the market represents the receiver, and social media disclosures constitute the communication channel through which strategic signals are transmitted. This framework naturally motivates the representation of executive signaling and market attention as time-varying probability distributions, denoted as $P_{t}$ and $Q_{t}$, respectively.

A critical challenge in operationalizing this framework lies in the high dimensionality and semantic sparsity of raw textual data, which precludes the direct construction of comparable distributions for the source and the receiver. To address this issue, we establish a shared semantic coordinate system by projecting both executive disclosures and market attention onto a reduced semantic basis. Topic saliency measures derived from the LDA model are employed to extract the top 30 most informative keywords [20]. These keywords collectively capture the core semantic structure of the digital opinion field while removing redundant and low-information terms. This semantic basis thus provides a common latent support on which both $P_{t}$ and $Q_{t}$ are defined. Formally, we define this shared semantic space as a discrete support set $\Omega =\{w_{1},w_{2},\ldots ,w_{K}\}$, where each element represents a distinct semantic unit in the discourse.

Based on the LDA topic modeling results outlined in Section 4.1.2, the Top K most significant keywords (where K=30) are extracted to form the semantic basis vector $\nu =\{w_{1},w_{2},\ldots ,w_{K}\}$. This basis set represents the core narrative focus of the digital opinion field during the observation period. For each trading day t, both the CEO’s social media behavior and the public’s search behavior are mapped onto this semantic basis to generate two corresponding probability distribution vectors. Specifically, the Market Attention Vector, denoted as $Q_{t}$ is derived from the search index associated with each keyword. Let $S_{t,i}$ represent the search volume for keyword i on day t. To ensure the vector constitutes a valid probability distribution, normalization is applied as shown in (15):

(15) $$Q_{t,i}=\frac{S_{t,i}+\epsilon }{\sum_{j=1}^{K}\left(S_{t,j}+\epsilon \right)},$$

where ϵ is a smoothing term introduced to prevent zero-division errors. Thus, $Q_{t}$ characterizes the actual distribution of market cognitive focus across the latent topics. Crucially, by satisfying the normalization constraint ($\sum Q_{t,i}=1$), $Q_{t}$ functions as a valid discrete probability distribution. This transformation decouples the structural allocation of attention from the absolute magnitude of search volume, providing a standard state for entropy calculation.

Simultaneously, the Executive Signal Vector, denoted as $P_{t}$, is constructed through a proposed neural-weighted projection mechanism. Within the communication-theoretic framework, $P_{t}$ represents the intended signal emitted by the information source. Let $c_{t,i}$ denote the frequency of keyword i in the CEO’s posts on day t. The weighted signal energy $E_{t,i}$ is defined by integrating the interaction metrics and sentiment scores derived in Section 4.1.3 and Section 4.1.1, respectively, as presented in (16):

(16) $$E_{t,i}=c_{t,i}\times (1+\ln(1+I_{t}))\times (1+B_{t}).$$

Here, $I_{t}$ represents the daily aggregate interaction volume (encompassing likes, comments, and reposts), and $B_{t}\in [0,1]$ is the daily average BERT sentiment score. The logarithmic transformation is employed to dampen the scale effect of extreme viral events while preserving the monotonicity of social influence. Subsequently, the signal energy vector is normalized to form the probability distribution $P_{t}$, as shown in (17):

(17) $$P_{t,i}=\frac{E_{t,i}+\epsilon }{\sum_{j=1}^{K}\left(E_{j,i}+\epsilon \right)}.$$

Accordingly, the normalized vector $P_{t}$ also functions as a discrete probability distribution. It represents the CEO’s intended signal, quantifying the relative emphasis placed on each topic after accounting for the emotional and viral intensity of the posts.

Finally, to quantify the structural mismatch between the executive’s intended signal ($P_{t}$) and the market’s actual attention ($Q_{t}$), the Kullback–Leibler (KL) Divergence [8] is employed. The SRE index for day t is formally defined in (18):

(18) $$SRE_{t}=D_{KL}(P_{t}∥Q_{t})=\sum_{i=1}^{K}P_{t,i}\log\left(\frac{P_{t,i}}{Q_{t,i}}\right).$$

In this analytical framework, a value of $SRE_{t}$ approaching zero indicates semantic resonance, implying that the distribution of the CEO’s signal closely aligns with the market’s search behavior, thereby facilitating high-efficiency information transmission. Conversely, a high SRE value indicates information dissipation, reflecting a significant divergence where the executive’s output fails to align with or trigger corresponding market attention. By synthesizing the Topic Relevance Index, Sentiment Orientation Index, and Virality Diffusion Index, the SRE index establishes a multidimensional framework for quantifying digital leadership effectiveness. Consequently, it serves as a critical input for the subsequent explanatory analysis of stock price volatility.

## 5. Experiments

### 5.1. Data Collection and Preprocessing

Existing research has shown that large-scale, aggregated social media sentiment exhibits a statistically significant association with key market indicators and can serve as an effective proxy for market sentiment [2]. To investigate the mechanism linking CEO social media activity and stock price dynamics, this study selects Xiaomi Corporation and Tesla Inc. as representative cases and retrieves their stock price data from the GTA (CSMAR) database. For Xiaomi, we use daily closing prices from April 2024 to October 2024, while for Tesla, we use daily closing prices from 2 December 2020 to 2 December 2024.

It is important to note that, due to variations in data accessibility and privacy regulations across platforms, this study presents an inherent asymmetry in the observation time windows for the two CEOs. This disparity arises primarily from platform-specific constraints rather than methodological design, yet measures were taken to ensure the representativeness and analytical rigor of the samples.

First, for Lei Jun, owing to Weibo’s ‘six-month visibility’ restrictions and technical barriers to accessing historical archives, the observation window is objectively constrained by platform-level data accessibility rather than discretionary sampling choice. Accordingly, this study selects the longest continuous time window currently available, yielding 490 original posts. Despite the relatively short temporal span, Lei Jun’s high-frequency and stable posting behavior ensures sufficient information density and granularity, thereby preserving the statistical representativeness of the sample.

Second, regarding Elon Musk, given the relatively unrestricted historical data access on the X platform (formerly Twitter) and the significance of his acquisition event in 2022, this study deliberately selected an extended dataset spanning two years before and after the acquisition. This design choice is intended to capture infrequent but highly influential episodic events and to adequately characterize behavioral regularities surrounding structural shifts in his communication style.

Accordingly, the two CEOs are explicitly treated as complementary, theory-informed case studies rather than symmetric panels requiring identical time coverage. This framing reflects their distinct leadership styles and information-transmission mechanisms and provides a coherent basis for comparative interpretation.

To construct the research corpus, this study employed Python (version 3.10)-based web scraping techniques to systematically collect all original posts published by Lei Jun on Weibo and Elon Musk on X (formerly Twitter) during the observation period. This dataset serves as the foundation for the subsequent textual analysis and sentiment metric construction. Notably, Musk’s acquisition of Twitter on 27 October 2022 constitutes a significant institutional inflection point. Arguing that this event precipitated a structural shift in his discourse power and information dissemination mechanisms, this study utilizes the acquisition date as a cutoff to segment the four-year sample into two distinct phases for comparative analysis.

### 5.2. Analysis of STV Indicators

#### 5.2.1. Sentiment Orientation Index

In the course of the sentiment analysis of posts, sentiment scores were assigned to Musk’s 30,735 posts, covering a period of four years. In order to process Lei Jun’s posts, the Chinese version of BERT was employed, termed BERT-Base-Chinese, in order to assign sentiment scores to his 490 posts over a six-month period.

To evaluate the out-of-sample reliability and generalization performance of the sentiment classification models, an additional validation procedure was conducted based on manually annotated data. Specifically, 40 posts were randomly sampled from Lei Jun’s Weibo corpus, and 100 posts were randomly sampled from Elon Musk’s posts in each of the pre-acquisition and post-acquisition periods. All sampled texts were independently labeled through blind human annotation, where a binary sentiment label was assigned, with 1 indicating positive sentiment and 0 indicating neutral or negative sentiment. The manually assigned labels were then compared with the model-predicted sentiment outcomes using a classification threshold of 0.5. The resulting accuracy and error rates are reported in Table 1.

Overall, the results demonstrate that the sentiment analysis models exhibit stable and satisfactory out-of-sample performance across different platforms and time periods, supporting their suitability for subsequent empirical analysis.

#### 5.2.2. Topic Relevance Index

**Figure 3:**
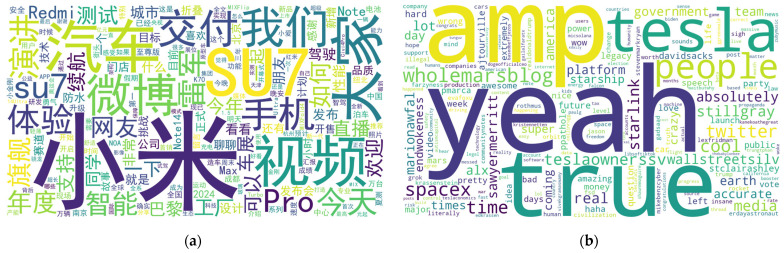
(**a**) Lei Jun’s post word cloud map; (**b**) Musk’s post word cloud map.

We first conducted a word frequency analysis on 491 posts published by Lei Jun between April and October 2024, as well as 6589 posts published by Elon Musk from 2 December 2020 to 27 October 2022, and visualized the results using word clouds, as shown in Figure 3 and Table 2.

The preliminary word frequency analysis reveals that function words or context-dependent terms, such as “我们 (we),” “大家 (everyone),” “cool,” and “time,” appear with high frequency in the text. However, these terms do not carry explicit thematic or sentiment information. Directly removing such high-frequency words based on predefined dictionaries or manual rules may partially disrupt the original semantic structure of the text, thereby affecting the accurate representation of the CEOs’ intended messages. Accordingly, this study does not rely on simple frequency-based filtering as the main analytical approach. Instead, Latent Dirichlet Allocation (LDA) is employed for topic modeling of the post corpus, enabling the identification of latent topic distribution patterns while preserving the overall semantic structure.

In the model specification process, the selection of the number of topics directly affects the model’s information extraction capacity and semantic interpretability. To address this, we adopt Perplexity and Topic Coherence as evaluation metrics, balancing statistical goodness-of-fit with semantic interpretability [30]. It should be noted that, in practical implementation, the perplexity measure is computed based on the log-likelihood of the held-out data. Specifically, perplexity is obtained through exponential transformation of the negative log-likelihood, i.e., Perplexity = $2^{-Log_{-}Likelihood}$. As a result, the intermediate Log-Likelihood values reported during model evaluation are negative by construction. For clarity of numerical presentation and to facilitate comparison across different topic specifications, this study reports Log-Likelihood values directly in Table 3, Table 4 and Table 5 as a monotonic transformation of perplexity, while preserving the same model selection criterion.

In this study, the LDA model is applied to the post corpora of Lei Jun (from 8 April 2024 to 7 October 2024) and Elon Musk across two distinct periods: the pre-acquisition phase (from 2 December 2020 to 27 October 2022) and the post-acquisition phase (from 28 October 2022 to 2 December 2024). Based on a rigorous iterative evaluation process, the results indicate that the optimal number of topics (K) is eight for Lei Jun’s posts, ten for Musk’s posts in the pre-acquisition period, and twelve for those in the post-acquisition period. These topic numbers correspond to the configurations under which the model attains the lowest perplexity and the highest topic coherence on the test set, thereby ensuring both the statistical reliability and the semantic interpretability of the topic modeling outcomes. The specific variations in these evaluation metrics corresponding to different topic numbers are detailed in Table 3, Table 4 and Table 5.

Following the determination of the optimal topic count, we extracted the top 30 keywords for each topic to characterize the semantic structure and thematic focus of the posts posted by Lei Jun and Elon Musk. Drawing on prior literature suggesting that integrating topic keywords with public search intensity serves as a valid proxy for investor attention and a potential precursor to market volatility [31], we adopted a similar approach.

**Figure 4:**
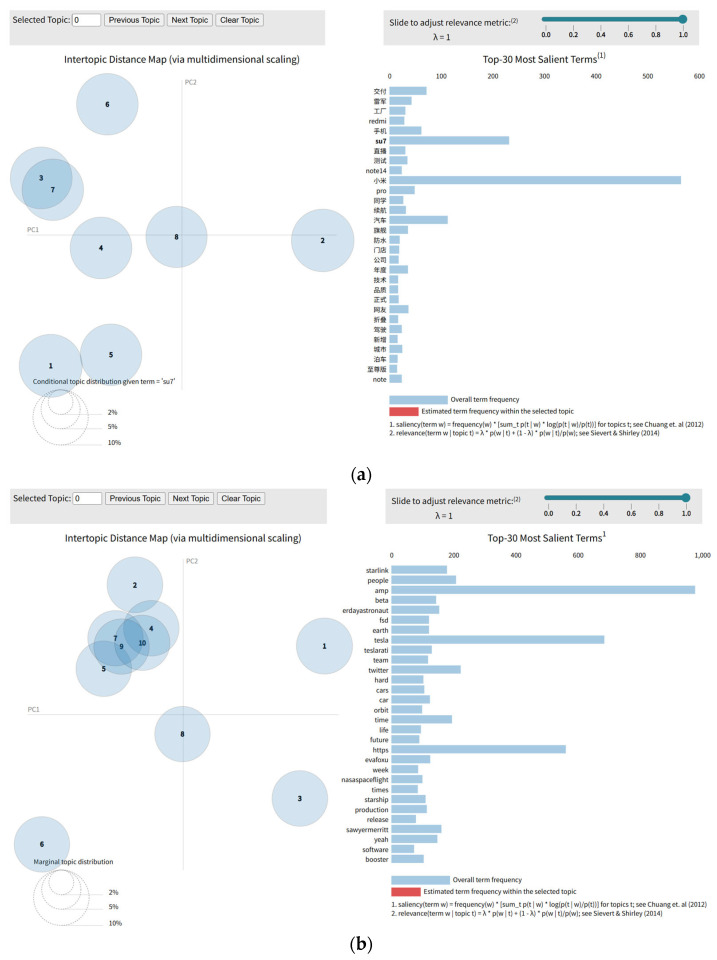

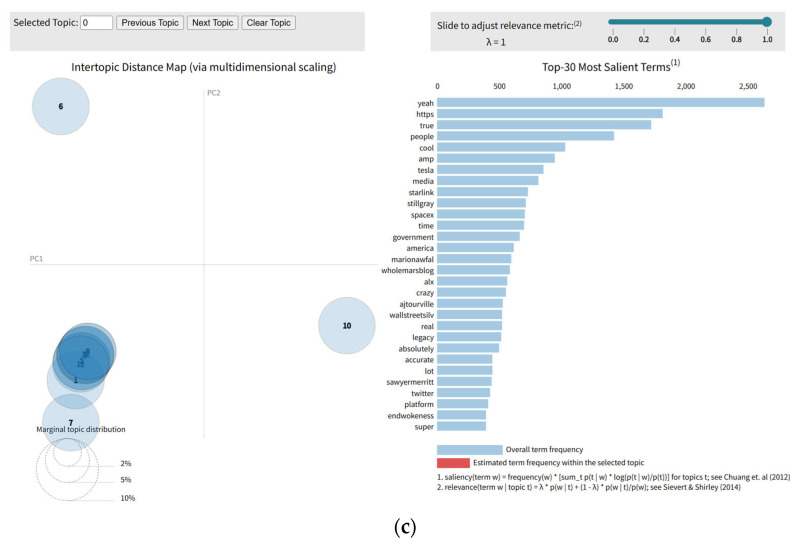
(**a**) Inter-Topic Distance Map for Lei Jun’s Posts; (**b**) Inter-Topic Distance Map and Top-30 Keywords for Elon Musk’s Posts (Pre-Acquisition); (**c**) Inter-Topic Distance Map for Elon Musk’s Posts (Post-Acquisition). ^1^ The Top-30 Most Salient Terms shown in this figure are calculated based on saliency [32]. ^2^ The LDA analysis interface shown in this figure is generated using the LDAvis visualization tool [33].

Building on this framework, we mapped the top 30 keywords from each topic to their respective search data. Specifically, search data for Lei Jun’s corpus was retrieved from the Baidu Search Index, while data for Elon Musk’s corpus was sourced from Google Trends. To ensure data consistency, keywords absent from these databases were excluded. The remaining terms were retained as valid empirical proxies for constructing the topic-related indices. The top 30 keywords extracted via the LDA model, along with their corresponding topic categories, are depicted in Figure 4 and Table 6.

#### 5.2.3. Virality Diffusion Index

**Figure 5:**
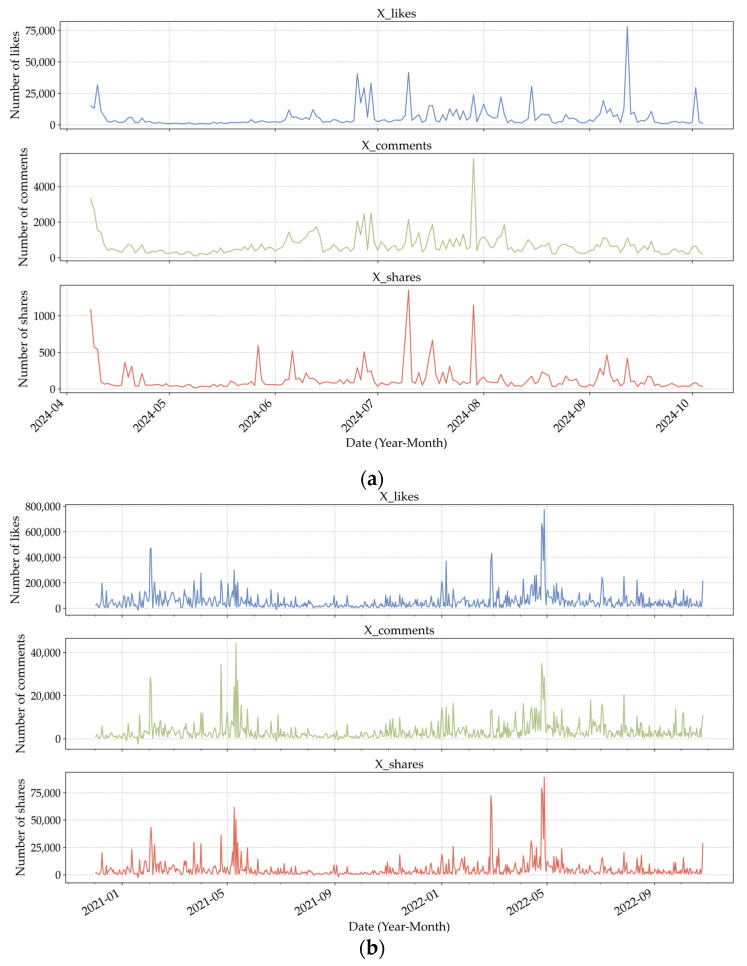

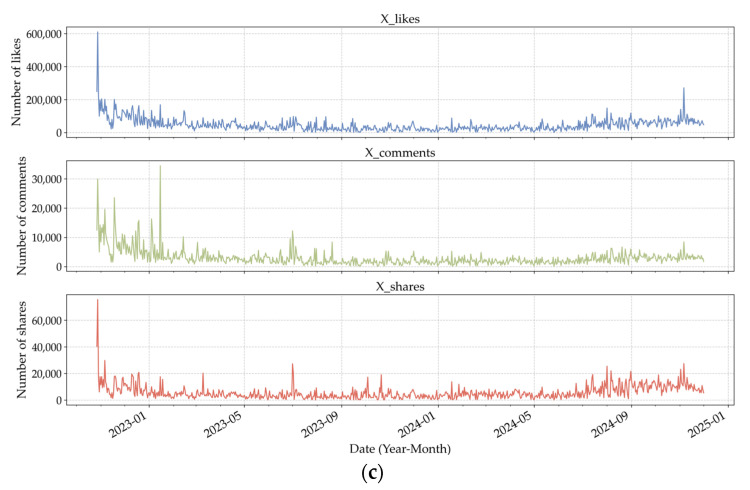
(**a**) Virality Diffusion Index for Lei Jun’s Posts; (**b**) Virality Diffusion Index for Elon Musk’s Posts (Pre-Acquisition); (**c**) Virality Diffusion Index for Elon Musk’s Posts (Post-Acquisition).

Building on the three dimensions discussed above, this study develops a multi-level evaluation framework for assessing the social influence of CEO posts. Within this framework, repost volume captures the breadth and coverage of information dissemination, the number of comments reflects the level of market attention and discussion intensity, and the number of likes reveals the prevailing emotional inclination of the audience. The Virality Diffusion Index for Lei Jun and Elon Musk is depicted in Figure 5.

### 5.3. Stock Price Prediction Results

**Figure 6:**
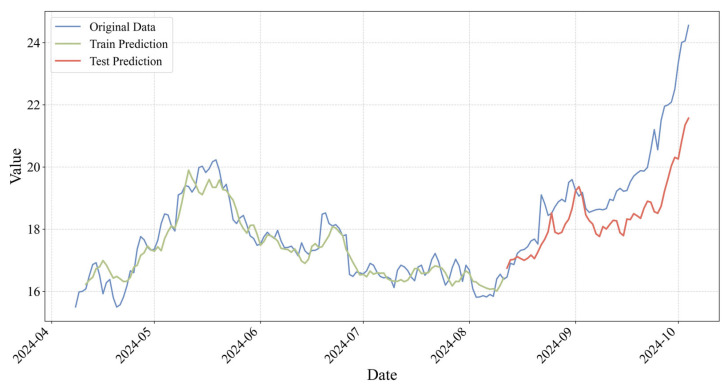
LSTM-Based Stock Price Prediction for Xiaomi.

After completing the training and hyperparameter optimization of the deep learning model, this study generates stock price prediction series for both the training and testing samples, and applies an inverse normalization procedure to restore the model outputs to the original price scale for performance evaluation. For Xiaomi Group (Figure 6), when the three-dimensional feature system constructed from Lei Jun’s social media activity is used as the model input, the LSTM model demonstrates a high level of fitting accuracy and generalization capability. The empirical results indicate that the Mean Absolute Error (MAE) remains at a relatively low level for both the training and testing sets, at 0.3156 and 0.9514, respectively. From the visualization perspective, the predicted price trajectory in the testing set (red solid line) closely aligns with the realized stock price series (blue solid line). The model not only accurately captures the long-term trend of stock price movements but also exhibits strong responsiveness at local turning points, including both peaks and troughs.

These findings suggest that for CEOs characterized by transactional leadership traits, the sentiment, thematic content, and interaction-based indicators embedded in their social media communications contain market signals with a high signal-to-noise ratio. Such signals can be effectively extracted by deep learning models and translated into accurate forecasts of firm valuation trajectories, providing evidence that information disclosure under this leadership style exhibits relatively strong market efficiency and predictability.

**Figure 7:**
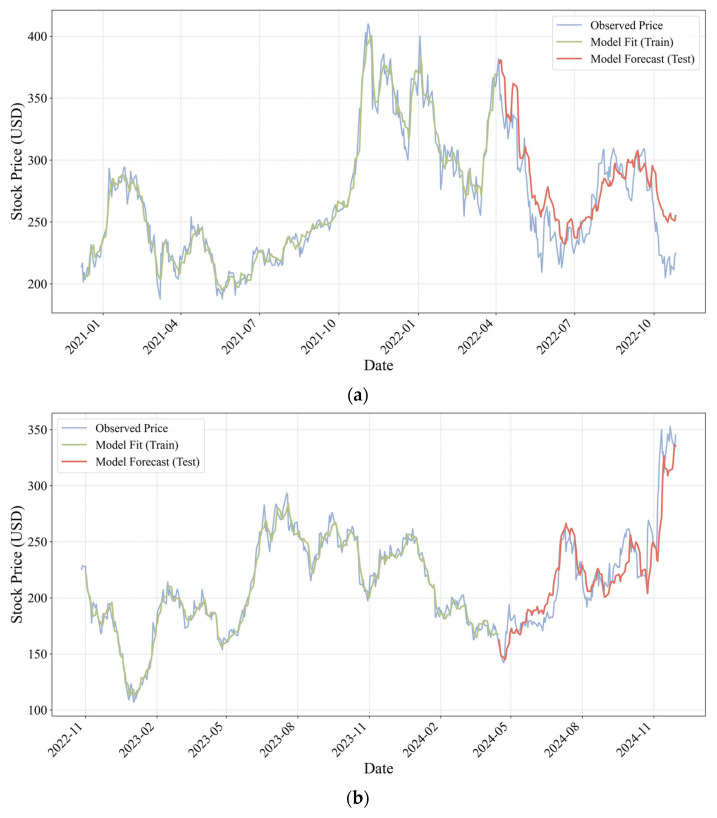
(**a**) LSTM-Based Stock Price Predictions for Tesla (Pre-Acquisition); (**b**) LSTM-Based Stock Price Predictions for Tesla (Post-Acquisition).

In contrast, the stock price prediction results for Tesla exhibit a markedly heterogeneous pattern (Figure 7). Although the model remains capable of capturing the overall fluctuation trend of the stock price, its predictive accuracy is substantially lower than that observed in the Xiaomi sample, and pronounced structural differences emerge across different stages. Empirical evidence shows that the Mean Absolute Error (MAE) for Tesla stock price prediction based on Musk’s posts is 22.95 in the pre-acquisition period, while the MAE decreases to 14.78 following the completion of the Twitter acquisition. This quantitative shift indicates that the informational coupling between Musk’s digital footprint and market valuation has notably strengthened in the post-acquisition period, effectively reducing the component of unexplained variance in the model’s output despite the asset’s inherent volatility.

Overall, the magnitude of prediction errors in the Musk sample is considerably higher than that in the Lei Jun sample, suggesting that the highly personalized and aggressive communication style associated with transformational leadership introduces substantial stochastic disturbances that are difficult for conventional deep learning architectures to smooth or regularize. However, a closer comparison between the pre- and post-acquisition phases reveals a form of structural recovery in predictive performance after the Twitter acquisition, as reflected by the notable decline in the MAE metric. This pattern of marginal improvement indicates that although Musk’s radical personal expression continues to generate post signals with a relatively high noise level, the completion of the acquisition and privatization of the social media platform appears to coincide with a more deliberate use of personal influence as a controllable instrument of digital governance. Enhanced control over the communication channel may have partially constrained the disorderly diffusion of information, allowing the mapping between post-based signals and capital market responses to evolve from an initially chaotic regime toward a more structured one. As a result, the explanatory power of the model improves in the post-acquisition period.

The comparative analysis above reveals fundamental differences across leadership styles. Specifically, even under an identical feature engineering pipeline and model architecture, the stock price predictability associated with CEOs’ social media behavior remains distinct. As a representative transactional leader, Lei Jun adopts a stable and pragmatic communication pattern. This approach constructs an information environment with high certainty, enabling predictive models to exhibit strong stability. By contrast, Elon Musk, as a prototypical transformational leader, relies on a highly personalized and often radical mode of expression, which appears to introduce a degree of randomness or structural noise into the information transmission process. As post-based signals propagate toward the capital market, this noise may lead to dissipation or distortion, weakening the direct mapping between semantic output and price dynamics.

Importantly, the divergence in predictive performance driven by leadership style cannot be simply attributed to differences in model specification or parameter calibration. Instead, it is more plausibly rooted in fundamental heterogeneity in the micro-physical structure of information diffusion, shaped by distinct leadership-driven communication regimes.

### 5.4. Semantic Resonance Dissipation Entropy Analysis

Although the LSTM model presented above exhibits strong nonlinear fitting capability in capturing stock price fluctuations, a core empirical anomaly remains insufficiently explained: why does the same “Sentiment–Topic–Social” (STV) multidimensional feature system, built upon an identical model architecture, deliver exceptionally high accuracy and robustness when predicting the stock price of Lei Jun (Xiaomi), yet display pronounced lag and bias when applied to Elon Musk (Tesla)? This contrast suggests that CEO digital leadership cannot be fully characterized along a purely quantitative dimension. While deep learning models are capable of learning complex input–output mappings, they fundamentally operate as “black boxes” and are unable to reveal how information qualitatively transforms as it propagates from the “CEO sender” to the “market receiver.”

To address this limitation, the present study introduces Semantic Resonance Dissipation Entropy (SRE), an interdisciplinary metric designed to overcome the explanatory constraints of conventional econometric approaches. From the perspective of information theory and complex systems, CEOs’ posts should not be treated merely as static textual observations, but rather as injections of “semantic energy flows” into the market system. Crucially, this injection process is not necessarily lossless. Informational “friction” emerges when the semantic focus intended by the CEO (represented by distribution (P) becomes misaligned with the attention focus actually generated by the market (represented by distribution (Q). This misalignment leads to the dissipation of semantic energy during transmission.

The SRE metric formalizes this mechanism by quantifying the distributional divergence between intention and market cognition, thereby capturing the effective signal-to-noise ratio embedded in social media communication. Within this framework, differences in leadership style are reflected in how CEOs structure and transmit information. Some communication strategies produce relatively low informational dissipation, facilitating efficient semantic alignment, whereas others generate higher levels of dissipation due to fragmented or diffuse market interpretation. By explicitly measuring this divergence, SRE provides an interpretable link between machine-learning-based predictive outcomes and management-theoretic mechanisms. More broadly, it offers a theoretically grounded approach to characterizing cognitive risk in asset pricing, particularly in environments where digital communication plays a central role in shaping investor behavior.

**Figure 8:**
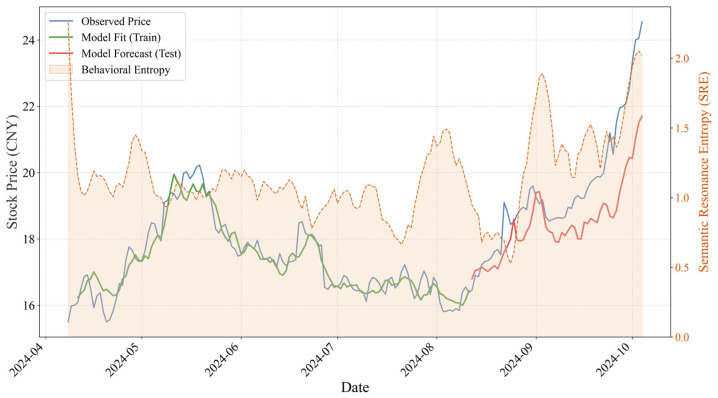
Co-movement of Stock Price and Semantic Resonance Dissipation Entropy (SRE) for Lei Jun (Xiaomi).

Figure 8 illustrates the temporal evolution of SRE for Lei Jun, representing a prototypical transactional leadership style. From a numerical perspective, Lei Jun constructs an exceptionally stable low-entropy information field, with SRE values persistently confined to a narrow range between 0.0 and 1.5, punctuated only by occasional pulse-like fluctuations at specific nodes. This low-baseline structure indicates that Lei Jun’s pragmatic, product- and technology-focused communication style generates a high degree of overlap between post semantics (P) and market search attention (Q), thereby substantially reducing friction in the information transmission process. More importantly, Lei Jun’s SRE exhibits the properties of a positive leading indicator for stock price movements. As shown in Figure 8, during the period from late August to September 2024, SRE displays a distinct and isolated peak, rising rapidly from approximately 0.5 to around 2.0. Notably, this increase in entropy does not reflect informational disorder; rather, it corresponds to a deliberate release of strategic signals—specifically, developments related to the SU7—when monthly deliveries first surpassed the threshold of 10,000 units. Following a brief phase of concentrated market attention, a valuation consensus formed swiftly, subsequently driving the stock price into a structurally upward trajectory in October.

This sequential transmission process, in which strategic signal disclosure is followed by a transient rise in entropy, subsequent convergence of market expectations, and eventual stock price appreciation, provides a rigorous explanation for the LSTM model’s ability to achieve high-precision trend identification in the Lei Jun sample.

**Figure 9:**
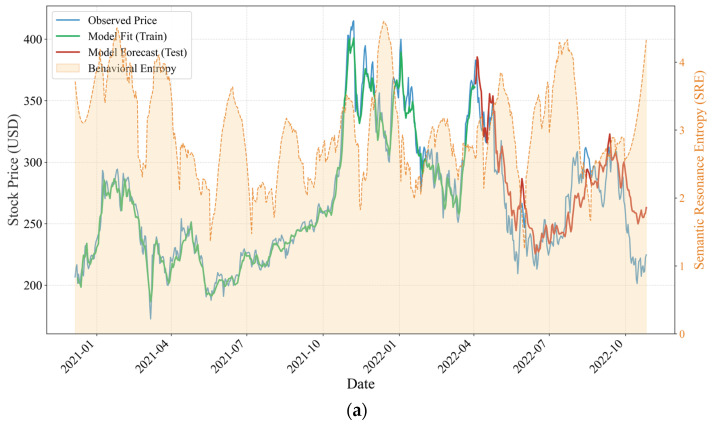

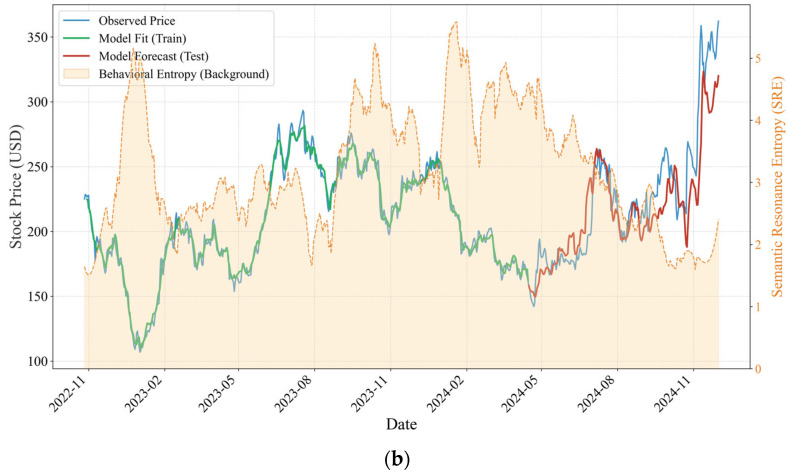
(**a**) Co-movement of Stock Price and Semantic Resonance Dissipation Entropy (SRE) for Elon Musk (Tesla: Pre-Acquisition); (**b**) Co-movement of Stock Price and Semantic Resonance Dissipation Entropy (SRE) for Elon Musk (Tesla: Post-Acquisition).

By contrast, the Musk sample, representing a transformational leadership style, exhibits a fundamentally different entropy profile. As shown in Figure 9a,b, Musk’s SRE values persistently remain within a high range of approximately 2.0 to 4.5, indicating an information environment characterized by intrinsically high dissipation and elevated noise levels. However, a comparison between the pre- and post-acquisition periods of Twitter reveals a clear shift in the underlying information transmission mechanism, evolving from a state of “unstructured chaos” toward one of “structural constraint.”

During most of the pre-acquisition period (Figure 9a, 2021 to early 2022), Musk’s SRE exhibits intense volatility with little discernible regularity, and its association with stock price movements is extremely weak. This random-walk–like entropy pattern suggests that posts during this stage failed to form an effective pricing factor, thereby preventing the LSTM model from learning a stable mapping between social media signals and market outcomes, which in turn resulted in relatively low predictive accuracy. Following the completion of the platform acquisition (Figure 9b), however, a statistically meaningful and stable inverse relationship between SRE and Tesla’s stock price emerges. Specifically, during the first and third quarters of 2023, episodes in which SRE surged above 4.0 were frequently accompanied by downward pressure and local troughs in Tesla’s stock price. Conversely, periods in which SRE retreated toward a lower level around 2.0 coincided with pronounced rebound windows. This pattern indicates that, for a transformational CEO, excessively high entropy no longer functions as a positive signal but instead manifests as a form of “governance risk” and “cognitive friction.” In such contexts, the market must incur substantial processing costs to filter noise embedded in aggressive public statements, thereby exerting negative pressure on prices. At the same time, Musk’s acquisition of the platform effectively strengthened the transmission efficiency of his digital leadership. Although the resulting effects may at times take the form of adverse governance signals, this risk has shifted from being an unobservable source of random noise to a market-discernible structural factor that can be priced.

When jointly considering the dynamic evolution of SRE and the predictive performance of the LSTM model, the structural divergence in entropy patterns provides micro-level evidence explaining the differentiation in forecasting effectiveness across the two samples. As a representative transactional leader, Lei Jun maintains a persistently low and stable SRE regime, thereby constructing a high signal-to-noise information environment in which semantic output closely aligns with market attention. This communication style reduces the complexity of market information processing and enhances both the accuracy and robustness of price predictions. In contrast, as a representative transformational leader, Musk exhibits a high-entropy, oscillatory SRE profile. Although high entropy persists after the acquisition, the emergence of a stable inverse suppression mechanism enables the market to more effectively hedge against noise-related risks. The LSTM model successfully captures this structural transition from disorder to an ordered negative association, which explains the marked improvement in predictive performance during the post-acquisition period. These findings provide strong evidence that transformational CEOs, by enhancing control over communication channels, can mitigate ineffective information dissipation and transform personal influence into a predictable asset pricing variable.

More broadly, this empirical evidence reveals a mechanism through which leadership style affects asset pricing efficiency by shaping the entropy properties of the information environment. Transactional leadership tends to generate an “entropy-reducing” effect by strengthening the alignment between semantic output and market attention, thereby lowering uncertainty and enhancing price predictability. Transformational leadership, by contrast, sustains high levels of attention but is more likely to induce an “entropy-increasing” effect, raising the cost of information processing for market participants. Consequently, in markets increasingly dominated by algorithmic trading, the stability of information entropy constitutes a critical boundary condition determining whether executives’ social media behavior can be effectively converted into market value creation dynamics.

### 5.5. Empirical Study

#### 5.5.1. Sensitivity Analysis

To examine whether the predictive performance of the LSTM component is sensitive to the choice of temporal lag structure, we conduct a sensitivity analysis by varying the time-step length used in the LSTM model. This analysis is motivated by the high-frequency nature of posts: CEO posts are released instantaneously, and market reactions tend to materialize over very short horizons. Consequently, short-term historical information is expected to play a dominant role in capturing the immediate price responses induced by social media activity.

In the baseline specification, the LSTM time-step length is set to 3. To assess robustness, we extend the time-step length to 5 and 7, thereby allowing the model to exploit relatively longer historical dependencies. The sensitivity analysis is implemented separately for Lei Jun and Elon Musk. For Elon Musk, the sample is further divided into two subsamples corresponding to the pre-acquisition and post-acquisition periods surrounding Musk’s takeover of Twitter. The predictive performance of each specification is evaluated using the mean absolute error (MAE), and the results are reported in Table 7 .

As shown in Table 8, for Lei Jun, the LSTM model with a time-step length of 3 achieves the lowest MAE. Increasing the time-step length to 5 leads to a marginal deterioration in predictive accuracy, while further extending the time-step length to 7 results in a substantial increase in prediction error. This pattern suggests that incorporating excessively long historical trajectories may obscure the high-frequency information embedded in CEO social media activity.

Consistent evidence is observed for Elon Musk. As reported in Table 9 for the pre-acquisition period and Table 10 for the post-acquisition period, the baseline specification with a time-step length of 3 consistently outperforms alternative lag structures. In both subsamples, longer time-step lengths of 5 and 7 are associated with higher MAE values, indicating inferior predictive performance relative to the short-horizon specification.

Notably, the performance gap across alternative time-step lengths is particularly pronounced in the post-acquisition period. As shown in Table 10, the MAE corresponding to a time-step length of 3 is markedly lower than those obtained under time-step lengths of 5 and 7. This finding implies that, following the Twitter acquisition, market participants responded more rapidly and more intensely to Musk’s social media activity, rendering short-horizon information especially informative for price formation. In contrast, incorporating longer historical dependencies appears to dilute the signal conveyed by high-frequency disclosures, thereby weakening predictive accuracy.

To further assess the robustness of the predictive results beyond single-run performance, this study conducts an extended robustness evaluation incorporating cross-validation, statistical hypothesis testing, and confidence interval estimation. Specifically, five-fold cross-validation is applied to each dataset to evaluate the stability of predictive accuracy across different data partitions. For each fold, the model is re-estimated and evaluated using mean absolute error, and the reported results correspond to the cross-validated mean and standard deviation.

In addition, to formally assess whether the predictive accuracy of the proposed LSTM model differs significantly from benchmark models, the Diebold–Mariano test is employed under the null hypothesis of equal forecast accuracy. Finally, a nonparametric bootstrap procedure is used to construct 95 percent confidence intervals for the MAE differences between LSTM and alternative models, providing a distributional assessment of estimation uncertainty.

The combined results of cross-validation, statistical testing, and confidence interval analysis are reported in Table 11. As shown, the LSTM model consistently achieves lower mean MAE across datasets, with statistically significant improvements over benchmark models in most cases. These findings provide strong evidence that the reported predictive advantage of the LSTM model is robust and not driven by a single-run realization or overfitting artifacts.

To mitigate concerns regarding potential overfitting from the concatenation of multi-dimensional feature vectors, we note that the LSTM model is implemented using the PyTorch library with multiple regularization strategies. Dropout regularization is applied both within the LSTM layers and as an additional layer to prevent co-adaptation of neurons, while L2 weight decay is used in the optimizer to penalize large model parameters. To further evaluate the necessity of preserving all feature dimensions, we compared the predictive performance of the LSTM model with and without dimensionality reduction via principal component analysis (PCA), retaining 90% variance. As reported in Table 12, the model using all concatenated features consistently outperforms the PCA-reduced input in terms of mean absolute error (MAE), indicating that the multi-dimensional features enhance prediction accuracy rather than introducing overfitting.

#### 5.5.2. Placebo Test

To further assess whether the predictive performance of the LSTM-based framework is driven by genuine temporal alignment between social media features and stock price dynamics, rather than mechanical correlations or model overfitting, we conduct a placebo test based on randomized time matching.

Specifically, for the three empirical settings, namely Lei Jun, Elon Musk before the Twitter acquisition, and Elon Musk after the acquisition, we preserve the internal structure of the feature vectors while randomly permuting their temporal correspondence with stock prices. The randomization is implemented at the daily level, such that the original within-day feature composition remains intact, while the alignment between features and contemporaneous prices is disrupted. In practice, this procedure is carried out in NumPy using np.random.shuffle(array) to reshuffle the temporal order of the feature–price pairs.

This design ensures that any predictive signal arising purely from the distributional properties of the features is retained, while the economically meaningful timing relationship between posts and market responses is intentionally destroyed. Under this placebo setting, we expect the model’s predictive accuracy to deteriorate substantially if the baseline results indeed rely on correct temporal matching. The predictive performance under the randomized alignment is reported in Table 13.

As shown in Table 12, the prediction accuracy declines sharply across all three datasets once the temporal correspondence between features and prices is randomized. Relative to the baseline specifications, the mean absolute errors increase markedly, indicating a significant loss of predictive power. This pattern is consistent across Lei Jun, Elon Musk before the acquisition, and Elon Musk after the acquisition, suggesting that the model’s forecasting ability critically depends on the correct timing of social media information rather than spurious correlations.

#### 5.5.3. Benchmark Comparison

To evaluate the relative predictive performance of the LSTM model and to rule out the possibility that the empirical results are driven solely by the choice of model, we further conduct a benchmark comparison. Specifically, three commonly used prediction methods—ARIMA, support vector regression (SVR), and back propagation neural network (BPNN)—are employed as alternative models and compared against the LSTM model under identical data and estimation settings.

The benchmark comparison is conducted separately for three datasets: Lei Jun, Elon Musk before the Twitter acquisition, and Elon Musk after the acquisition. All models are trained on the same training samples and evaluated over the same prediction windows, with mean absolute error (MAE) used as the common metric for predictive accuracy. This approach ensures comparability across different models and allows for a direct assessment of whether the LSTM model achieves superior predictive performance.

**Figure 10:**
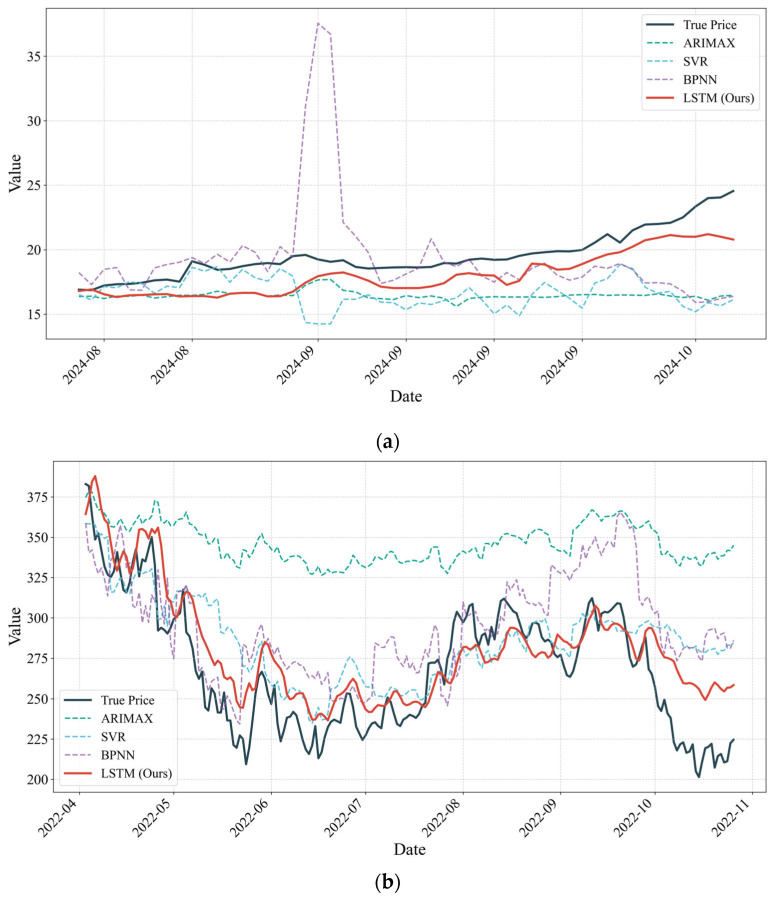

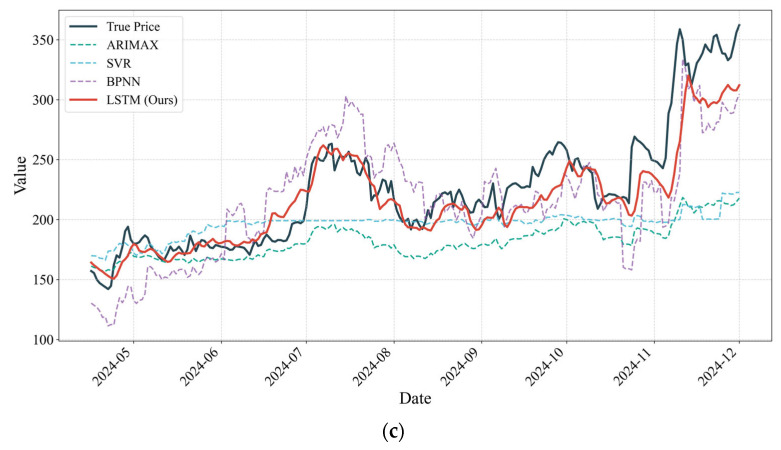
Comparison of Predicted and Realized Stock Prices across Alternative Models (**a**) Predictions for Lei Jun; (**b**) Predictions for Elon Musk before the Twitter acquisition; (**c**) Predictions for Elon Musk after the Twitter acquisition.

The MAE results for all models are summarized in Table 14, and the corresponding predicted versus realized stock price paths are illustrated in Figure 10.

As shown in Table 13, the LSTM model consistently achieves the lowest MAE across all three datasets, outperforming ARIMA, SVR, and BPNN. This indicates that LSTM exhibits superior predictive accuracy, particularly in capturing complex nonlinear relationships and high-frequency dynamic features. By contrast, traditional time-series models and shallow machine learning models are comparatively less effective in tracking these patterns.

The results depicted in Figure 10 further demonstrate that the LSTM model more accurately follows both the overall trend and local fluctuations in stock prices, exhibiting smaller deviations from realized prices. The predictive advantage of LSTM is especially pronounced in samples characterized by event-driven dynamics, suggesting that the model is better equipped to handle nonlinear shocks induced by social media activity.

## 6. Conclusions

This study develops an interdisciplinary analytical framework grounded in information diffusion theory, market reaction theory, and strategic leadership theory to examine how CEOs’ social media behavior drives the dynamic evolution of firm market value under different leadership styles. Using Lei Jun of Xiaomi Group and Elon Musk of Tesla as representative cases, the analysis combines high-frequency post data with capital market data to investigate the informational mechanisms linking executive communication to stock price dynamics. The study constructs a multidimensional STV feature system capturing sentiment orientation, thematic relevance, and social penetration, which is used as input to an LSTM-based stock price prediction model. To address the interpretability limitations of machine-learning-based predictions, the study proposes a novel metric, Semantic Resonance Dissipation Entropy, grounded in information theory and physics. This metric quantifies the temporal and structural misalignment between executive semantic output and market attention, providing micro-level evidence on how leadership style influences asset pricing efficiency.

Based on this framework, the empirical results confirm that deep learning models utilizing multidimensional social media features are effective in forecasting short-term stock price dynamics. However, their predictive efficacy is fundamentally contingent upon the informational structure established by leadership style. Transactional leadership fosters a stable environment of low entropy where executive semantics align closely with market attention. Within this context, occasional increases in entropy serve as valid indicators of strategic business transitions and exert a positive influence on firm valuation. Conversely, transformational leadership exhibits a pattern of high entropy and information dissipation, where aggressive and personalized communication often generates cognitive friction. The analysis reveals that heightened entropy under this style correlates negatively with stock price performance, acting as a suppressive factor driven by informational disorder. Consequently, the Semantic Resonance Dissipation Entropy metric offers a structural explanation for these divergences, highlighting that the economic value of digital leadership depends on the ability of executives to actively manage their digital influence to ensure signal clarity and prevent the generation of noise that induces volatility.

The findings highlight that the economic value of executive social media activity is fundamentally conditioned by information structure rather than message volume or sentiment alone. For corporate leaders, effective digital communication requires alignment between leadership style and information governance, as stable semantic output reduces market uncertainty, while excessive entropy can translate into pricing discounts. For regulators, executive control over high-impact communication channels introduces structural risks that cannot be adequately addressed through content-based supervision alone, calling for monitoring frameworks that capture attention misalignment and entropy dynamics. For investors, the results suggest that social media signals should be evaluated through their informational coherence with market attention rather than through surface-level emotional intensity, as only low-entropy or structurally constrained high-entropy environments yield signals that are consistently relevant for asset pricing.

## Figures and Tables

**Table 1:** Out-of-Sample Sentiment Classification Performance.

	Lei Jun	Musk 2020–2022	Musk 2022–2024
**Accuracy (%)**	87.5	81.0	92.0
**Error Rate (%)**	12.5	19.0	8.0

**Table 2:** Lei Jun’s post word cloud map: Chinese–English Comparison (Top 10).

Chinese	English
小米	Xiaomi
汽车	car
大家	everyone
视频	video
我们	we
微博	Weibo
交付	delivery
手机	phone
演讲	speech
今天	today

**Table 3:** LDA Indicators of Lei Jun’s Posts.

Number of Topics	Log-Likelihood	Theme Consistency
2	−8.5051	0.28
4	−8.5632	0.3426
6	−8.5714	0.3552
8	−8.576	0.4254
10	−9.6143	0.4333
12	−21.5178	0.423
14	−27.497	0.4419
16	−28.1346	0.451
18	−28.1377	0.4524

**Table 4:** LDA Indicators of Elon Musk’s Posts (Pre-Acquisition).

Number of Topics	Log-Likelihood	Theme Consistency
2	−28.6982	0.2417
4	−31.8078	0.2801
6	−32.7293	0.3633
8	−33.2794	0.3847
10	−34.4522	0.4337
12	−35.1246	0.4423
14	−35.2986	0.4828
16	−35.4671	0.5018
18	−35.7900	0.5283

**Table 5:** LDA Indicators of Elon Musk’s Posts (Post-Acquisition).

Number of Topics	Log-Likelihood	Theme Consistency
2	−43.0150	0.2679
4	−43.4725	0.3370
6	−43.7018	0.3569
8	−43.9968	0.3750
10	−44.3176	0.4223
12	−44.5113	0.4667
14	−44.6207	0.4716
16	−44.8310	0.4838
18	−45.0525	0.5001

**Table 6:** LDA Top 30 Keywords: Chinese-English Correspondence.

Chinese	English
小米	Xiaomi
汽车	car
雷军	Lei Jun
工厂	factory
直播	live streaming
测试	test
同学	classmate
续航	range
旗舰	Flagship
防水	waterproof
年度	annual
技术	technology
品质	quality
正式	official
网友	netizen
折叠	collapse
驾驶	drive
新增	add
城市	city
泊车	parking
至尊版	premium edition
交付	delivery
手机	phone
门店	storefront
公司	company

**Table 7:** Comparison of MAE results on training and testing sets for Lei Jun and Elon Musk.

Subject	Training MAE	Testing MAE
**Lei Jun**	0.2718	1.4072
**Musk (Pre-Acquisition)**	6.6670	18.9735
**Musk (Post-Acquisition)**	5.3681	13.4399

**Table 8:** Sensitivity of LSTM Prediction Accuracy to Time-Step Length: Lei Jun.

Time Steps (Lag)	MAE Score
3 (Base)	1.029
5	1.033
7	2.062

**Table 9:** Sensitivity of LSTM Prediction Accuracy to Time-Step Length: Elon Musk (Pre-Acquisition Period).

Time Steps (Lag)	MAE Score
3 (Base)	23.1644
5	25.7124
7	24.1636

**Table 10:** Sensitivity of LSTM Prediction Accuracy to Time-Step Length: Elon Musk (Post-Acquisition Period).

Time Steps (Lag)	MAE Score
3 (Base)	14.7421
5	21.7478
7	19.6393

**Table 11:** Robustness Evaluation Based on Five-Fold Cross-Validation, Statistical Tests, and Confidence Intervals.

Dataset	Model	Mean MAE (Std. Dev.)	DM Test *p*-Value (vs. LSTM)	95% CI of MAE Difference
**Lei Jun**	LSTM (Base)	0.91 (0.53)	–	–
	SVR	1.03 (0.61)	0.0015	[0.041, 0.191]
	BPNN	1.11 (0.39)	0.0530	[0.015, 0.396]
	ARIMAX	1.54 (0.87)	<0.001	[0.517, 0.751]
**Musk 2020–2022**	LSTM (Base)	29.03 (14.86)	–	–
	SVR	30.39 (13.06)	0.0538	[0.132, 2.765]
	BPNN	61.40 (50.99)	<0.001	[25.48, 40.33]
	ARIMAX	44.63 (18.79)	<0.001	[13.77, 17.47]
**Musk 2022–2024**	LSTM (Base)	23.75 (10.06)	–	–
	SVR	24.28 (11.35)	0.5920	[−1.34, 2.64]
	BPNN	50.88 (25.57)	<0.001	[19.78, 35.37]
	ARIMAX	40.25 (11.14)	<0.001	[14.32, 18.71]

**Table 12:** Comparison of LSTM Prediction Accuracy between Full Feature Concatenation and PCA-Reduced Features.

Configuration	Lei Jun (MAE)	Musk 2020–2022 (MAE)	Musk 2022–2024 (MAE)
**Original**	1.4072	18.9735	13.4399
**PCA (90%)**	1.6374	23.7632	18.6452

**Table 13:** Placebo Test Based on Randomized Time Alignment between Features and Stock Prices.

Model	Lei Jun (MAE)	Musk 2020–2022 (MAE)	Musk 2022–2024 (MAE)
**Original Model**	1.03	23.16	14.74
**Placebo Model**	2.54	44.44	47.48

**Table 14:** Predictive Performance Comparison across Benchmark Models.

Model	Lei Jun (MAE)	Musk 2020–2022 (MAE)	Musk 2022–2024 (MAE)
**ARIMA**	3.0654	77.7306	43.7646
**SVR**	2.9101	24.2861	36.6188
**BPNN**	2.8156	32.5041	30.0301
**LSTM**	1.5117	19.4510	15.8395

## Data Availability

The tweet data of Lei Jun and Elon Musk, with all associated code generated during the current study, have been deposited in the Hugging Face repository and will be made publicly available at a link to be provided upon acceptance.

## Abbreviations

The following abbreviations are used in this manuscript:

CEO	Chief Executive Officer
STV	Sentiment–Topic–Virality
BERT	Bidirectional Encoder Representations from Transformers
LDA	Latent Dirichlet Allocation
LSTM	Long Short-Term Memory
SRE	Semantic Resonance Dissipation Entropy
KL	Kullback–Leibler
ARIMA	AutoRegressive Integrated Moving Average
SVR	Support Vector Regression
BPNN	Back Propagation Neural Network
GARCH	Generalized Autoregressive Conditional Heteroskedasticity
DCC	Dynamic Conditional Correlation
